# Stem cell-derived co-grafts contribute to retinal reconstruction and visual functional improvement in a laser damaged rat model

**DOI:** 10.1186/s40779-025-00601-7

**Published:** 2025-05-21

**Authors:** Deepthi S. Rajendran Nair, Magdalene J. Seiler, Juan Carlos Martinez-Camarillo, Yuntian Xue, Ruchi Sharma, Kapil Bharti, Mark S. Humayun, Biju B. Thomas

**Affiliations:** 1 Department of Ophthalmology, USC Roski Eye Institute, University of Southern California, Los Angeles, CA 91030 USA; 2 Departments of Physical Medicine and Rehabilitation, Ophthalmology, Anatomy, and Neurobiology, University of California, Irvine, Irvine, CA 92697 USA; 3 Stem Cell Research Center, University of California, Irvine, Irvine, CA 92697 USA; 4 USC Ginsburg Institute for Biomedical Therapeutics, University of Southern California, Los Angeles, CA 90033 USA; 5 Biomedical Engineering, University of California, Irvine, Irvine, CA 92617 USA; 6 Unit on Ocular and Stem Cell Translational Research, National Eye Institute, NIH, Bethesda, MD 20892 USA

**Keywords:** Stem cell-based therapy, Retinal organoids, Retina laser damage, Retinal degenerative disease, Retinal pigment epithelium, Retinal transplantation, Co-graft

Dear Editor,

Irreversible retinal damage can occur due to retinal degenerative (RD) diseases as well as injuries caused by accidents or devices. Laser devices can inflict permanent damage to the retina, leading to the loss of photoreceptors (PRs) and underlying retinal pigment epithelium (RPE), culminating in vision impairment. Since there is no effective treatment for permanent retinal injuries, replacing damaged PRs and RPE with corresponding healthy cells can be a suitable therapeutic approach.

Although various transplantation approaches were demonstrated in congenital RD disease models [1, 2], for the first time we used a novel tissue-engineered co-graft made of RPE and retinal organoid (RO) sheets (Fig. 1; Additional file 1: Figs. S1, S2) to treat laser-damaged retinal injuries (Fig. 1a; Additional file 1: Materials and methods). Athymic nude rat retinas were exposed to green diode laser (IRIDEX IQ 532) photocoagulation (50–80 mW) in the left eyes in an area approximately the size of the co-grafts (0.4 mm × 0.9 mm) (Additional file 1: Fig. S3). Athymic nude rats were used to minimize the possible immunological issues related to the use of human derived RPE and RO sheets for transplantation. Co-grafts were made of human embryonic stem cell (hESC) derived RO sheets dissected from 90 to 110-day-old ROs (Fig. 1b, f; Additional file 1: Figs. S1, S2) and induced pluripotent stem cell (iPSC)-derived RPE cells grown as a monolayer on vitronectin-coated ultrathin parylene membrane [3] (Fig. 1c, d, f). Previously, we had used different bioadhesives to combine RPE monolayer and RO sheets, including matrigel, gelatin, Col-Tgel, and medium viscosity G (MVG) alginate [4]. Considering the rigidity and adhesion property constraints associated with these materials, we used a new approach here based on fibrin glue as the bioadhesive [5]. The co-grafts were transplanted into the subretinal space of athymic nude rats in the laser-damaged area using a specially designed tool (Fig. 1e) that enabled precise graft placement (Fig. 1f–h). Post-surgical optical coherence tomography (OCT) imaging confirmed the subretinal placement (Fig. 1j) of the grafts and its growth and maturation were monitored until 7 months. Visual functional benefits were measured periodically using optokinetic nystagmus (OKN) testing and electroretinogram (ERG) recording. About 7-months post-surgery, electrophysiological mapping of the brain visual center, the superior colliculus (SC), was conducted as a terminal procedure followed by histological examinations.

**Fig. 1:**
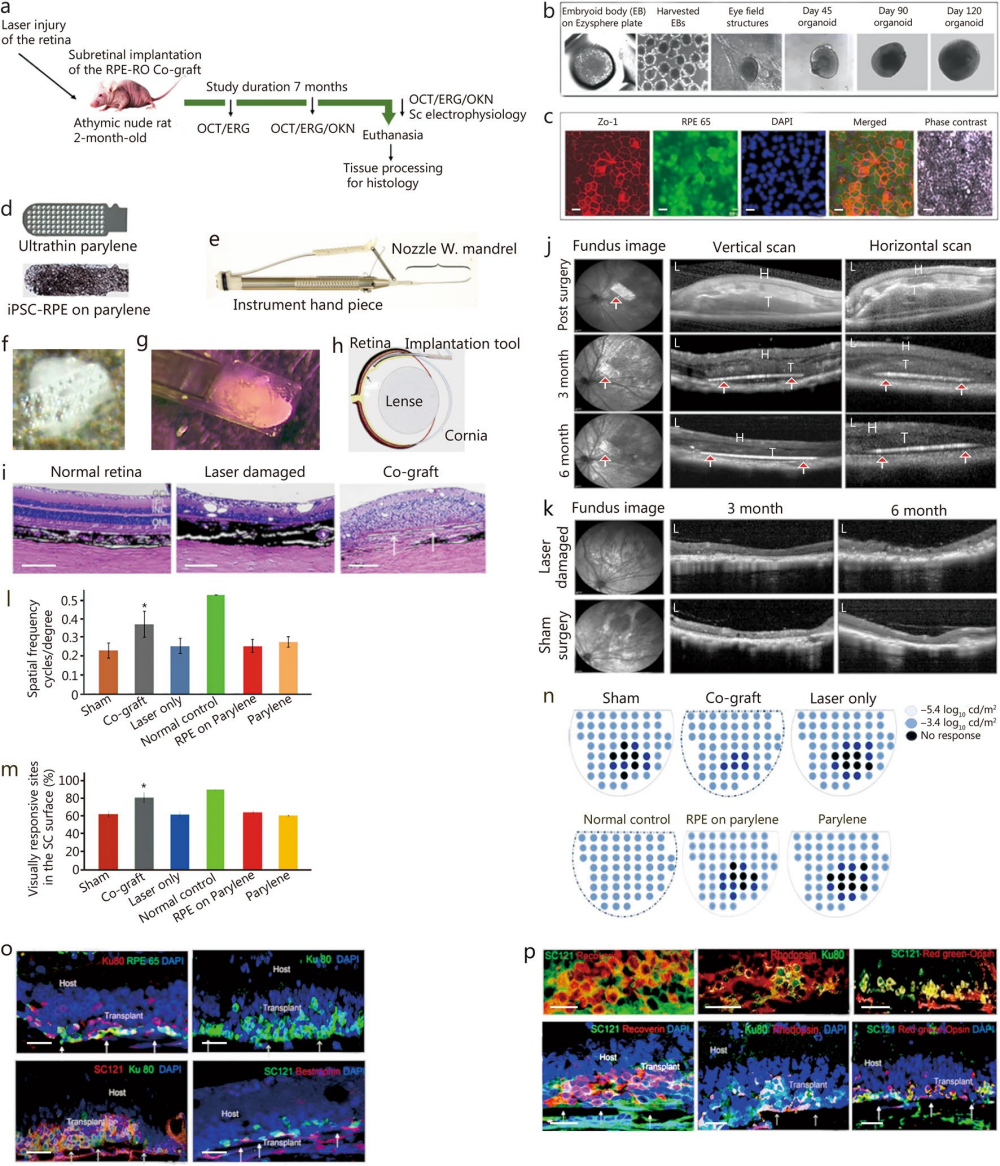
Stem cell-derived co-grafts contribute to the retinal reconstruction and visual functional improvement in laser damaged athymic nude rat model. **a** Schematic protocol of the experimental procedures in athymic nude rats with retinal laser injury. **b** Various stages of retina organoid (RO) development-embryoid body on EZSPHERE plate, harvested embryoid bodies in 100 mm plate, eye field structures developed in matrigel coated plates, and ROs at different developmental stages. **c** Induced pluripotent stem cell-derived retinal pigment epithelium (iPSC-RPE) expressing different RPE markers: zonula occludens-1 (Zo-1), retinal pigment epithelium-specific 65 kD protein (RPE 65), nuclear stain DAPI, and phase contrast image of iPSC-RPEs. Scale bar = 50 µm. **d** Ultrathin parylene membrane before and after culturing of iPSC-RPE monolayer **e** Co-graft implantation tool. **f** Co-graft made of iPSC-RPE and RO sheet using fibrin glue. **g** Co-graft loaded into the nozzle of the implantation tool prior to transplantation. **h** Diagrammatic sketch showing subretinal implantation of the co-graft. **i** Hematoxylin and eosin (HE) stained image of the athymic nude rat retinas. **j** Optical coherence tomography (OCT) imaging of the retina-fundus images showing the co-graft in the vertical, and horizontal scans immediately after the surgery, 3-months post-surgery, and 6-months post-surgery. Hyperreflexion in the OCT images (white arrows with red arrowhead) indicates the subretinal placement of the parylene membrane. H host, T transplant. **k** Fundus image and OCT scanning image of the laser damaged and sham surgery retina showing the retinal thinning that remained unchanged over time. Scale bar = 200 µm. **l** Optokinetic nystagmus (OKN) testing conducted at 6 month time points. Significant visual improvement in co-graft implanted rats compared to the laser-damaged control rats (*P* < 0.05) was evident at 6 month time point. *P < 0.05. **m** Superior colliculus (SC) activities recorded by single electrode mapping showing statistically significant (*P* < 0.05, Student’s *t*-test) improvement in visual responses in the co-graft implanted rats compared to the sham surgery group and laser damaged control group. *P < 0.05 **n** SC activities recorded by multielectrode array (MEA) mapping at 7 months post-surgery. Laser beams caused a focal retinal injury that resulted in the absence of any visual activity (scotoma) that was partially repaired by co-graft implantation. **o** Confocal images of the immunostained athymic nude rat retinas 7 months post-surgery. Transplanted co-grafts expressed RPE 65 (RPE marker)/DAPI, Ku80 (human nuclear marker)/DAPI, SC121 (human cytoplasmic marker/Ku80/DAPI, and bestrophin (RPE marker)/Ku80. White arrows denote parylene membrane. **p** Transplanted co-grafts expressing general photoreceptor marker, recoverin, rod specific marker rhodopsin and cone photoreceptor marker, red-green opsin. Scale bar = 100 µm

Histology analysis of laser-injured eyes (Fig. 1i; Additional file 1: Fig. S3) showed significant damage to multiple retinal layers (most importantly PR and RPE). Vertical and horizontal OCT scan images corroborated evidence of suitable graft placement (Fig. 1j). Distinct structural modifications in the appearance of the co-graft were noticed at various post-surgical time points. Compared to the immediate post-surgery OCT imaging showing a raised and thick appearance, the co-graft appeared more intact at 3- and 6-month (Fig. 1j) time points. In the laser-damaged control rat groups, sham surgery rat groups (Fig. 1k), parylene implanted rat groups and RPE on parylene implanted rat groups (Additional file 1: Fig. S4), the thinning of the retina remained unchanged.

The ERG recording failed to detect apparent differences between various experimental groups (Additional file 1: Fig. S5). This can be because of the small size of the laser-damaged area. It is plausible that visual deficit is limited to such a very small area (less than 1 mm) may not be sufficient to cause measurable changes in the ERG signals. OKN measurements showed improvement in visual acuity in co-graft implanted groups compared to the sham group and the control laser-damaged groups at the 6-month time point (*P* < 0.05; Fig. 1l). Single electrode mapping of the SC demonstrated significantly higher number of SC sites responding to light in the co-graft implanted rats (*n* = 5) compared to the sham surgery group (*n* = 4) and the control laser-damaged rats (*n* = 2, *P* < 0.05; Fig. 1m). Corroborating the above observation, spatial properties of the visual responses measured using multielectrode array (MEA) (Fig. 1n; Additional file 1: Fig. S5) demonstrated the absence of scotoma in most of the co-graft implanted rats (3/4). In contrast, the presence of scotoma was evident in all sham surgery rats (*n* = 4), parylene only implanted rats (*n* = 1), parylene + RPE implanted rats (*n* = 4), and control laser damaged rats (*n* = 2; Fig. 1n).

Histological examination of the co-graft transplanted eyes demonstrated the presence of co-grafts containing RPE and RO entities. Immunostaining with human nuclear antigen (Ku80) and cytoplasmic marker (SC121) confirmed that the transplanted cells were of human origin (Fig. 1o). Differentiation of cells inside the co-graft into PRs was evidenced by the expression of the general PR marker recoverin, as well as specific markers for rods (rhodopsin) and cones (red-green opsin, Fig. 1p). Prevalence of rosette formation of transplanted PRs was mostly absent in these rats compared to our previous studies in which various congenital disease models were used [1, 2, 4]. This can be due to the improved co-graft fabrication technique, although differences between disease models may also play a role. The laser damaged control eyes showed a total absence of RPE and PRs in the laser damaged area 7 months after surgery (Additional file 1: Fig. S3).

The RPE cells in the co-graft in vivo expressed the RPE markers such as bestrophin and RPE 65 (Fig. 1o), suggesting their survival. Synaptophysin, a marker for presynaptic vesicles, exhibited strong staining in both host and transplant, suggesting possible synaptic connections between host and transplant (Additional file 1: Fig. S6). Staining targeting cellular retinaldehyde binding protein, which is specific for Müller cells and RPE, demonstrated the presence of Müller cells within the transplants (Additional file 1). Additionally, the host’s Müller cells exhibited a greater degree of reactivity compared to those in the transplant, as indicated by glial fibrillary acidic protein (GFAP) staining (Additional file 1: Fig. S7). Rod bipolar cells [marker: protein kinase C alpha (PKCα)] were also present in the transplant layer (Additional file 1: Fig. S7). Notably, there was no visible presence of fibrin glue, suggesting its complete degradation, enabling integration between the RPE and the organoid sheet. This study presents a more advanced method to create retinal co-grafts by combining RO sheet and polarized RPE monolayer, with fibrin glue serving as the bioadhesive. The survival and maturation of PRs in the co-graft after transplantation, along with the resulting improvements in visual function, render this technique appropriate for the treatment of permanent retinal injuries.

Co-graft implanted retinas require more extensive studies to better understand morphological integration, outer segment formation, and synaptic connectivity. While no rosette formation was observed, and the transplanted cells were notably preserved, the co-grafts did not fully develop the organized, laminar structure typical of normal retinas. The proximity of transplanted cells to intermediate neurons suggests potential for synaptic connections, although partial disorganization and loss of laminar structure were observed in histological analysis, likely due to the small eye size and xenograft issues in rat models. Further studies focusing on synaptic connectivity are essential, given that cytoplasmic transfer poses a risk associated with photoreceptor transplantation. However, unlike previous studies which used single-cell suspensions derived from primary or progenitor cells that may lead to cytoplasmic transfer [6], the integrity of the co-grafts in this study suggests that this effect is unlikely. Future use of focal electroretinography (ERG) could provide insight into functional differences between damaged and healthy retinal areas, contributing to improved transplant efficiency. This "proof of concept" in immunodeficient rat models marks a significant milestone for tackling advanced retinal degeneration diseases in humans.

This study demonstrates the therapeutic potential of co-grafts composed of retinal organoid (RO) sheets and retinal pigment epithelium (RPE) for retinal reconstruction. Using a laser-damaged rat model, we showed that co-graft implantation improves visual function, highlighting a promising approach for treating retinal injuries and degenerative diseases. The co-graft method, supported by fibrin glue as a bioadhesive, facilitates the survival, maturation, and functional integration of transplanted photoreceptors, enabling synaptic connections with host retinal neurons. Further research in larger animal models is essential to address immune rejection and optimize graft placement, but this work lays an important foundation for translating tissue-engineered retinal therapies into clinical applications targeting vision loss from retinal degeneration.

## Supplementary Information

**Additional file 1: Materials and methods. Table S1** List of primary antibodies. **Table S2** List of secondary antibodies. **Fig. S1** Characterization of CRX-GFP retinal organoids. **Fig. S2** Preparation of 3D retina organoids for transplantation. **Fig. S3** Optical coherence tomography (OCT) and immunohistochemistry (IHC) of control and laser damaged retinas. **Fig. S4** Optical coherence tomography (OCT) and hematoxylin and eosin (HE) staining of parylene and retinal pigment epithelium (RPE) on parylene implanted retinas. **Fig. S5** Electroretinogram (ERG) data and superior colliculus (SC) electrophysiology setup. **Fig. S6** Transplanted co-grafts expressing recoverin and synaptophysin. **Fig. S7** Transplanted Co-grafts expressing glial fibrillary acidic protein (GFAP)/Ku80 (human nuclear marker), and cellular retinaldehyde binding protein (CRALPB; specific for Müller cells and RPE)/protein kinase C alpha (PKCα; human specific-Rod bipolar cell marker) after 7 months.

## Data Availability

All data generated or analyzed during this study are included in this published article and its supplementary information file. Further inquiries are available from the corresponding author upon reasonable request.

## Abbreviations

CRX: Cone-rod homeobox gene
ERG: Electroretinogram
iPSC: Induced pluripotent stem cells
MEA: Multielectrode array
OCT: Optical coherence tomography
OKN: Optokinetic nystagmus
PR: Photoreceptors
RD: Retinal degenerative
RO: Retinal organoid
RPE: Retinal pigment epithelium
SC: Superior colliculus
