# Supplementary material for: Stem cell-derived co-grafts contribute to retinal reconstruction and visual functional improvement in a laser damaged rat model
Source: Mil Med Res. 2025 May 21;12:23. doi: 10.1186/s40779-025-00601-7 (PMC12093716; doi:10.1186/s40779-025-00601-7)
Supplement: Supplementary file 1 — Additional file 1: Materials and methods. Table S1 List of primary antibodies. Table S2 List of secondary antibodies. Fig. S1 Characterization of CRX-GFP retinal organoids. Fig. S2 Preparation of 3D retina organoids for transplantation. Fig. S3 Optical coherence tomography (OCT) and immunohistochemistry (IHC) of control and laser damaged retinas. Fig. S4 Optical coherence tomography (OCT) and hematoxylin and eosin (HE) staining of parylene and retinal pigment epithelium (RPE) on parylene implanted retinas. Fig. S5 Electroretinogram (ERG) data and superior colliculus (SC) electrophysiology setup. Fig. S6 Transplanted co-grafts expressing recoverin and synaptophysin. Fig. S7 Transplanted Co-grafts expressing glial fibrillary acidic protein (GFAP)/Ku80 (human nuclear marker), and cellular retinaldehyde binding protein (CRALPB; specific for Müller cells and RPE)/protein kinase C alpha (PKCα; human specific-Rod bipolar cell marker) after 7 months. [file 40779_2025_601_MOESM1_ESM.pdf]

Materials and methods

Ethics statement

All experiments were approved by the University of Southern California Institutional Animal Care and Use Committee (IACUC, Protocol No. 21303) and were performed following the National Institutes of Health (NIH) Guide for the Care and Use of Laboratory Animals and the Association for Research in Vision and Ophthalmology (ARVO) Statement for the Use of Animals in Ophthalmic and Vision Research.

Animals

Forty immunodeficient NIH nude rats Charles River Laboratories (Wilmington, MA) were used to create the retinal laser injury model. These rats have deficient T cell activity with lymph nodes and peyers patches showing lymphocyte depletion in T-lymphocyte dependent regions. The rats were kept in ventilated cages with food and water ad libitum, and were included in the study after they reached the age of 60 d postnatal (P), and then were divided into the following 6 groups: co-graft implanted (n = 9), sham surgery (n = 8), control laser damaged (n = 4), parylene + retina pigment epithelium (RPE) (n = 9), parylene only (n = 3) and no surgery control (n = 4). Three rats that showed corneal abnormalities or surgical complications were excluded from the final study.

Human RPE derived from induced pluripotent stem cells (iPSCs)

iPSC-RPE cells were obtained in trans well inserts from Dr. Kapil Bharti (Unit on Ocular and Stem Cell Translational Research, National Eye Institute, NIH, Bethesda, USA). iPSCs were generated by reprogramming healthy adult human fibroblasts. Briefly, iPSCs were cultured on the vitronectin-coated surface in E8 media. The cells were passaged at 70 – 80% confluency. For RPE differentiation, iPSCs were seeded at a density of (2 – 4) $\times 10^5$/well of a 6-well plate. Cells were fed every day with specific induction and committed to RPE fate. The RPE committed stage (d 25) and immature RPE stage (d 40) were checked for maturity and purity using Flow assay for RPE-specific markers.

Culturing of polarized iPSC-RPE monolayer on parylene membrane

Immature iPSC-RPEs were seeded on vitronectin-coated parylene membranes and cultured in X VIVO 10 media (Lonza, Hayward, CA, USA) for 6 weeks. The maturity was confirmed by transepithelial electrical resistance (TER) measurement and RPE

maturity markers with medium changes thrice a week. The parylene substrates used consisted of an ultrathin membrane of parylene (0.30 µm thick) with a 6 µm thick supporting mesh developed by Regenerative Patch Technologies (RPT, CA, USA). The cells were allowed to grow to confluence for approximately four weeks before preparation of the co-graft.

Differentiating retinal organoids (ROs) from cone-rod homeobox-green fluorescent protein (CRX-GFP) H9 cells

NIH-registered H9 human embryonic stem cells (hESCs), genetically modified with a GFP tagged to the *CRX* gene (donated by Majlinda Lako, Newcastle University, UK), were used to differentiate retina organoids. The CRX-GFP cell line derived from H9 (NIH 0043) was cultured at the University of California, Irvine (UCI, Irvine, CA) and the University of Southern California (USC, Los Angeles, CA, USA), through a material transfer agreement with University of Newcastle, Biosciences Institute, UK. *CRX* encodes cone-rod homeobox protein and is specifically expressed in photoreceptor cells. CRX-GFP H9 cells were cultured in mTeSR 1 media (STEMCELL Technologies, Vancouver, BC, Canada) and passaged at 80% confluency using ReLeSR (STEMCELL Technologies, Vancouver, BC, Canada). Cells were expanded on BD GFR Matrigel-coated plates at 37 °C in a humidified 5% CO_2 incubator (Nuaire, Plymouth, MN, USA). For the differentiation of retina organoids, accutase (Nacalai Inc., Kyoto, Japan) was added to the confluent stem cell culture to make a single-cell suspension. The cells were then transferred to an 800-µm micro-well EZSPHERE 6-well plate (Nacalai USA, Inc., San Diego, CA, USA) and centrifuged at 100 × *g* for 3 min using a plate centrifuge. Embryoid bodies (EB) were formed from d 1 to d 7 in the EZSPHERE microwells. On d 8, the EBs were seeded onto a 1% growth factor reduced matrigel (Corning, NY, USA) coated culture dish. The media was gradually transitioned from mTeSR1 medium into neural induction media (NIM) containing Dulbecco's modified eagle medium (DMEM)/F12 (1:1) (Gibco, Waltham, MA, USA), 1% N_2 supplement (Gibco), 1× minimum essential media non-essential amino acids (NEAA) (Gibco), 1× L-glutamine (Gibco), and 2 µg/ml heparin (Sigma-Aldrich, St. Louis, MO, USA), with daily media changes. EB attach and spread into the culture dish and start differentiation into eye field structures. From d 19 to d 41, the media was switched to NIM containing DMEM/F12 (1:1) supplemented with 2% B27 supplement (50×) (minus vitamin A, Gibco), 1× NEAA, 1× L-glutamine, and 2 mg/ml heparin (Sigma, Burlington, USA). The retinal eye fields

were cut out from the dish between 40 – 50 d and transferred to ultra-low attachment 24-well plates (Corning, NY, USA). From d 19 to d 41, the media was switched to NIM containing DMEM/F12 (1:1) supplemented with 2% B27 supplement (50×) (minus vitamin A, Gibco, MT, USA), 1× NEAA, 1× L-glutamine, and 2 mg/ml heparin. From d 42 onwards, the organoids were cultured with media containing DMEM/F12 (1:1) supplemented with 2% B27 plus supplement (50×) (Gibco, MT, USA), 1× NEAA, 1× L-glutamine, 2 μg/ml heparin, 100 μmol/L taurine (Sigma, Burlington, USA), and 10% fetal bovine serum (Gibco, Montana, USA).

Preparation of co-grafts using RO sheets and RPE sheets

CRX-GFP labeled ROs aged between 90 – 110 d (average 105 d) when photoreceptor maturation is initiated were used in this study. ROs were selected based on the laminated structure and outer transparent layer with a hollow spherical shape as seen by phase contrast microscope. RO sheets of dimension averaging (0.7 – 1.3) mm × 0.6 mm were cut to make the co-graft. The RPE portion in the ROs was removed and the remaining RO sheet [(0.2 – 0.3) mm × 0.6 mm] was used to facilitate smooth entry of the co-graft into the surgical tool. Based on the outcome of the pilot studies, we used ≤ 8 μl of fibrin glue (Baxter Healthcare Corp, IL, USA) that promoted the adherence of co-graft. The surgical eye received additional treatment with betadine, followed by gentamycin/polymyxin/bacitracin ointment (Bausch & Lomb, Rochester, NY, USA). In earlier studies, various bioadhesives such as growth factor-reduced matrigel (1:2; Fisher Scientific, Waltham, MA, USA; or Corning, NY, USA), gelatin (1% – 5%; Sigma, St. Louis), Col-Tgel (3D Collagen gel, 101 Bio USA), and medium viscosity G (MVG) alginate (1%; Pronova, Oslo, Norway) were employed to merge the RPE monolayer and RO sheets. Due to limitations in rigidity and adhesion properties associated with these materials, the choice for the current study involved opting for fibrin glue as the bioadhesive. The cograft preparation was timed in parallel to the laser damage procedure and subretinal implantation was performed immediately after confirming the desired degree of laser damage (damage to the RPE and photoreceptors).

Retinal laser damage and subretinal transplantation

The animals were anesthetized with ketamine/xylazine (80 – 90 mg/kg, 5 – 10 mg/kg), and pupils were dilated with 1% atropine eye drops. The eye was disinfected with ophthalmic betadine (Alcon, Fort Worth, TX). The non-surgical eye was kept moist with the application of artificial tears. Rat retinas were exposed to green diode laser (IRIDEX

IQ 532) photocoagulation (50 – 80 mW) in the left eyes in an area approximately the size of the co-grafts. Since the size of each laser spot was approximately 75 μm, multiple spots were required to make injury close to the size of a rat implant (400 μm × 900 μm). The degree of laser damage was determined based on an immediate fundus examination and optical cutting medium (OCT) imaging.

For retinal transplantation, a small incision (around 1 mm) was made posterior to the pars plana, parallel to the limbus, followed by local retinal detachment. The transplants were delivered to the subretinal space of the left eye using a previously used custom-made delivery device. The surgical eye received additional treatment with betadine, followed by gentamycin/polymyxin/bacitracin ointment (Bausch & Lomb, Rochester, NY, USA). Rats were placed in a temperature-controlled incubator (Thermocare, Paso Robles, CA) for recovery. During anesthesia recovery, rats were placed on a heating pad and received subcutaneous injections of anti-sedan (atipamezole hydrochloride, Zoetis, NJ, USA) and the analgesic buprenorphine (buprenex, 0.03 mg/kg) for anesthesia recovery and pain management. The following rat surgery groups were used: co-graft implanted (n = 9), sham surgery (n = 8), parylene + RPE implanted (n = 9) and parylene only implanted (n = 3). All sham surgery rats received retinal laser injuries, surgical procedures, and medium-alone injections (without any tissue). A control group that received laser damage alone was also included (n = 4).

Spectral domain OCT (SD-OCT) imaging

The SD-OCT images of the retina were obtained using a spectralis Heidelberg Retina Angiography and Optical Coherence Tomography (HRA + OCT) device (Heidelberg Engineering, Heildeberg, Germany). SD-OCT imaging was used to document and monitor the placement of the transplant in the host retina. Transplanted rats were imaged at 1, 3 and 6 months after surgery. The animals were anesthetized with ketamine/xylazine, and the pupils were dilated using atropine. Basic scans of a 2.6 mm × 2.6 mm area were taken by including the optic disk. If the transplants are in the further periphery, additional scans were taken to include those areas. The 488 × 488 × 5 (# B-scans/#A-scans/B-scan averaging value) scans were used to obtain fundus images; then the same area was scanned by 800 × 20 × 80 or 700 × 70 × 25 scans to obtain B-scans. Using 488 × 488 × 5 scans, B-scan images were used to outline the transplant edges on corresponding fundus images. Then ImageJ was used to calculate the size (area) of the transplants. Rats with transplant misplacement or severe surgical distress (n = 2) were excluded from further

analysis after the first or second examination. The final OCT scan was scheduled close to the terminal experiment [superior colliculus (SC) electrophysiology].

Electroretinography (ERG)

ERG was performed at 3- and 6-months post-surgery using the HMsERG system (Ocuscience, Las Vegas, NV). For ERG assessment, animals that were dark adapted overnight were anesthetized as described previously along with the application of pupil dilation and topical anesthesia eye drops. A heating table was used to maintain the body temperature. Reference and ground electrodes were inserted into the infraorbital (malar) area and between the ears, respectively. Scotopic and photopic ERGs were recorded from both eyes, and the raw data were collected to assess the b-wave amplitude of the ERG waveforms.

Optokinetic nystagmus (OKN) testing

The visual acuity of athymic nude rats was measured by OKN testing at 3- and 6-months post-transplantation using a previously described protocol. Briefly, two tablet screens were used to display the high-contrast black and white stripes generated using "OKN Stripes Visualization Web Application", a freely available software (http://mdds.nyc/okn-stripes-visualization). The rat was partially restrained using a clear plexiglass holder, and their eyes were constantly exposed to the tablet screen. The rats were monitored for head tracking in the clockwise and anticlockwise direction of stripe rotations for 1 min per frequency. The responses were recorded at spatial frequencies 0.15, 0.20, 0.24, 0.28, and 0.33. Both the left and right eyes were tested by alternating the direction of the moving stripes. Head tracking responses were recorded using a mini camera attached to the top of the rat holder. Video recordings were evaluated to compute the head-tracking scores by two separate investigators who were blind to the experimental condition. Comparison between groups or between the two eyes (left eye vs. right eye) was conducted using a suitable statistical analysis. Three independent tests are performed at each time point and the highest visual acuity level was considered as the final visual acuity score.

SC electrophysiology

For SC electrophysiology, a previously established methodology was followed [4]. Briefly, rats were dark-adapted overnight and then sedated with intraperitoneal injection of xylazine/ketamine (5/37.5 mg/kg respectively) and maintained on 1 – 3% inhaled sevoflurane in medical oxygen. Pupils were dilated with 0.5% tropicamide and 2.5%

phenylephrine. All procedures were performed in a dark room with a dim red light. Body temperature was maintained by a heating pad. Rats were placed in a digital stereotactic apparatus (David Kopf Instruments, Tijunga, CA), right parietal craniotomy was performed with a handheld drill (Dremel, Walnut Ridge, AR), and a small amount of cortex overlying the right SC was aspirated to directly visualize the SC. The stereotactic apparatus was referenced from the lambda suture and used to guide the placement of a custom-made single tungsten microelectrode within the right SC. The reference electrode was placed near the exposed scalp and the ground electrode was placed subcutaneously in the tail region. Visual responses to a full-field flash of -1.6 $\log_{10}$ cd/m^2 (Grass PS 33 Photic stimulator, W. Warwick, RI) were recorded using the Powerlab data acquisition system (ADInstruments, Mountain View, CA, USA). Recordings were performed from 28 – 30 SC locations (200 – 400 μm apart) covering the fullest visible extent of the SC. Based on the SC activity, 3 – 10 trials were performed at each recording site. Using the stereotactic coordinates of the electrode penetrations, a graphical representation of the SC mapping data was plotted to determine the degree of visual activities in the co-graft implanted rats (n = 5) compared to the sham surgery group (n = 4), parylene only implanted rats (n = 2), parylene + RPE implanted rats (n = 5) and the control laser damaged rats (n = 2). For multielectrode array (MEA) mapping, our recently established technique was used [7]. For this, co-graft implanted (n = 4), sham surgery (n = 4), parylene only implanted (n = 1), parylene + RPE implanted (n = 4), control laser damaged rats (n = 2) and no surgery control rats (n = 2) were used. After the SC was exposed, the MEA was advanced into the superficial SC. The visual activities were recorded during full-field light stimulation of the left eye at two different light intensities ranging from -5.4 $\log_{10}$ cd/m^2 to -1.6 $\log_{10}$ cd/m^2. Recordings were made using a Lablynx 64-channel recording system (NeuraLynx, 105 Commercial Drive, Bozeman, MT 59715). Response distribution from all 56 MEA channels was plotted to map the visual activities in the SC.

Histology and immunohistochemistry (IHC)

For histology of ROs, 4% paraformaldehyde was used to fix the eyes for 20 min, followed by 3 washes in phosphate-buffered saline (PBS, STEMCELL Technologies, Vancouver, BC, Canada). After ROs were incubated overnight in 30% sucrose in PBS, embedded in OCT (CellPath, Powys, UK), and frozen at −20 °C. Ten micrometer cryostat sections were collected onto Super frost Plus slides and stored at −20 °C in slide boxes before immunostaining (see **Additional file 1: Table S1** for list of antibodies used). Before

immunostaining, cryosections were air-dried for 20 min at room temperature, hydrated with PBS for 30 min, and incubated with a blocking solution containing 10% goat serum and 0.3% Triton X-100 (Sigma-Aldrich, MA, USA) for 1 h at room temperature. Slides were incubated with the appropriate primary antibody overnight at 4 °C (a list of antibodies is included in **Additional file 1: Table S1**). After rinsing with PBS, sections were incubated with the secondary antibody for 2 h at room temperature (a list of antibodies is included in **Additional file 1: Table S2**). After washing, the tissue sections were counter-stained with Hoechst 33342 (Life Technologies, Rockville, MD, USA). Negative controls were carried out by omitting the primary antibody.

All animals after the completion of visual functional assessments (OKN and electrophysiology) were euthanized for histological assessments [hematoxylin and eosin (HE) and IHC] between 6 to 7 months post-surgery. For histology of the eyes, the rats were euthanized by intracardiac injection of euthasol (Virbac AH, Inc., Fort Worth, TX, USA), and the whole eyes were fixed using Davidson's solution (Electron Microscopy Sciences, PA, USA). After overnight incubation with Davidson's solution, the cornea and lens were removed followed by embedding eye cups in paraffin. 5-μm sections were taken before proceeding to staining. For light microscopy, every fifth slide was stained with hematoxylin and eosin. The HE-stained slides were scanned and photographed using an Aperio Scanscope CS (Aperio Technologies, INL, Vista, CA, USA) microscope. Histological sections of co-graft implanted retinae were evaluated to assess co-graft survival. The surgical placement was considered acceptable if 100% of the implant was located inside the subretinal area. Transplant survival was confirmed based on the presence of the co-graft in at least three consecutive sections and based on immunostaining evaluations. Details of the antibodies used are included in **Additional file 1: Table S1**. For immunostaining, all slides were deparaffinized using xylene and rehydrated using alcohol gradients. Sodium citrate, pH 6.0 (Electron Microscopy Sciences, PA, USA) was used for antigen retrieval. The tissue sections were incubated with a blocking solution containing 10% donkey serum and 0.3% Triton X-100 for 1 h at room temperature. Slides were incubated with the appropriate primary antibody overnight at 4 °C after staining, and the slides were mounted with fluorescent-enhanced mounting medium with 4′,6-diamidino-2-phenylindole (DAPI; Vector Laboratory, Burlingame, CA, USA). Images were taken using the Ultra viewer ERS dual-spinning disk confocal microscope (PerkinElmer, Waltham MA, USA) equipped with a C-Apochromat (Carl Zeiss, Thornwood, NY, USA) ×10 high dry lens, a C-Apochromat ×40 water immersion

lens NA 1.2, an electron multiplier charge-coupled device cooled digital camera (Hamamatsu Orce_ERCC 12-bit camera; PerkinElmer, Waltham, MA, USA). Images were captured and processed using ImageJ software (https://imagej.net).

Image acquisition and processing

Retinal sections were viewed on a Zeiss Axio ImagerZ2 equipped with Apotome 2 and Zen 2012 blue software (Carl Zeiss, Jena, Germany). Objectives lens used were EC Plan Neofluar ×20/0.5 Ph2, EC Plan Neofluar ×40/1.3 Ph2, EC Plan Apochromat ×63/1.4 Ph3. Series of XZ optical sections (< 1 μm thick) were taken at 1.0 μm steps throughout the depth of the section. Final images are presented as a maximum projection and adjusted for brightness and contrast in Adobe Photoshop CS6 (Adobe, San Jose, CA, USA).

Statistical analysis

Rats were randomized into age-matched control, parlene only control, RPE + parylene control, non-surgery laser-damaged control, sham surgery, and co-graft transplanted group. For all statistical analyses, the significance level was calculated in Graphpad Prism software (La Jolla, CA) with Analysis of Variance (ANOVA), and paired and unpaired *t*-tests using mean ± SEM. The level of significance was set at $P < 0.05$.

Table S1 List of primary antibodies

Antigen	Species	Specific for	Dilution	Supplier	Catalog #
Bestrophin	Mouse	RPE	1:200 – 1:500	Millipore	MAB5466
CRALBP	Rabbit	RPE and Muller cells	1:1000	Abcam	ab243664
Ku80	Rabbit	Human nuclei	1:400	Abcam	ab80592
Recoverin	Rabbit	Photoreceptors, cone bipolar cells	1:2K	Millipore	AB5585-I
Rhodopsin (rho1D4)	Mouse	Rods	1:100	Invitrogen	MA5-45247
RPE 65	Mouse	RPE	1:100	Novus	NB100-35
SC-121 (STEM121)	Mouse	The cytoplasm of human cells	1:2000	Stem Cell Inc.	AB-121-U-050
PKCα	Rabbit	Rod bipolar cells	1:100	Santa Cruz Biotechnology	sc-8393
Synaptophysin	Goat	Membrane protein of synaptic vesicles	1:100	Novus Biologicals	AF5555
GFAP	Mouse	Reactive glial cells	1:100	Cell Signaling Technology	#3670

CRALBP cellular retinaldehyde-binding protein, *Ku80* human nuclear antigen marker, *RPE 65* retinal pigment epithelium-specific 65 kD protein, *SC-121* human cytoplasm marker, *PKCα* protein kinase C alpha, *GFAP* glial fibrillary acidic protein

Table S2 List of secondary antibodies

Conjugate	Species	Specific for	Dilution	Supplier	Catalog #
Alexa Fluor 488	Donkey	Rabbit IgG (H + L)	1:400	Jackson ImmunoResearch, USA	711-545-152
Rhodamine Red-X	Donkey	Rabbit IgG (H + L)	1:400	Jackson ImmunoResearch	711-295-152
Alexa Fluor 488	Donkey	Mouse IgG (H + L)	1:400	Jackson, USA ImmunoResearch	715-545-150
Alexa Fluor™ 594	Donkey	Goat IgG (H + L)	1:400	Thermo Fischer Scientific, USA	A-11058

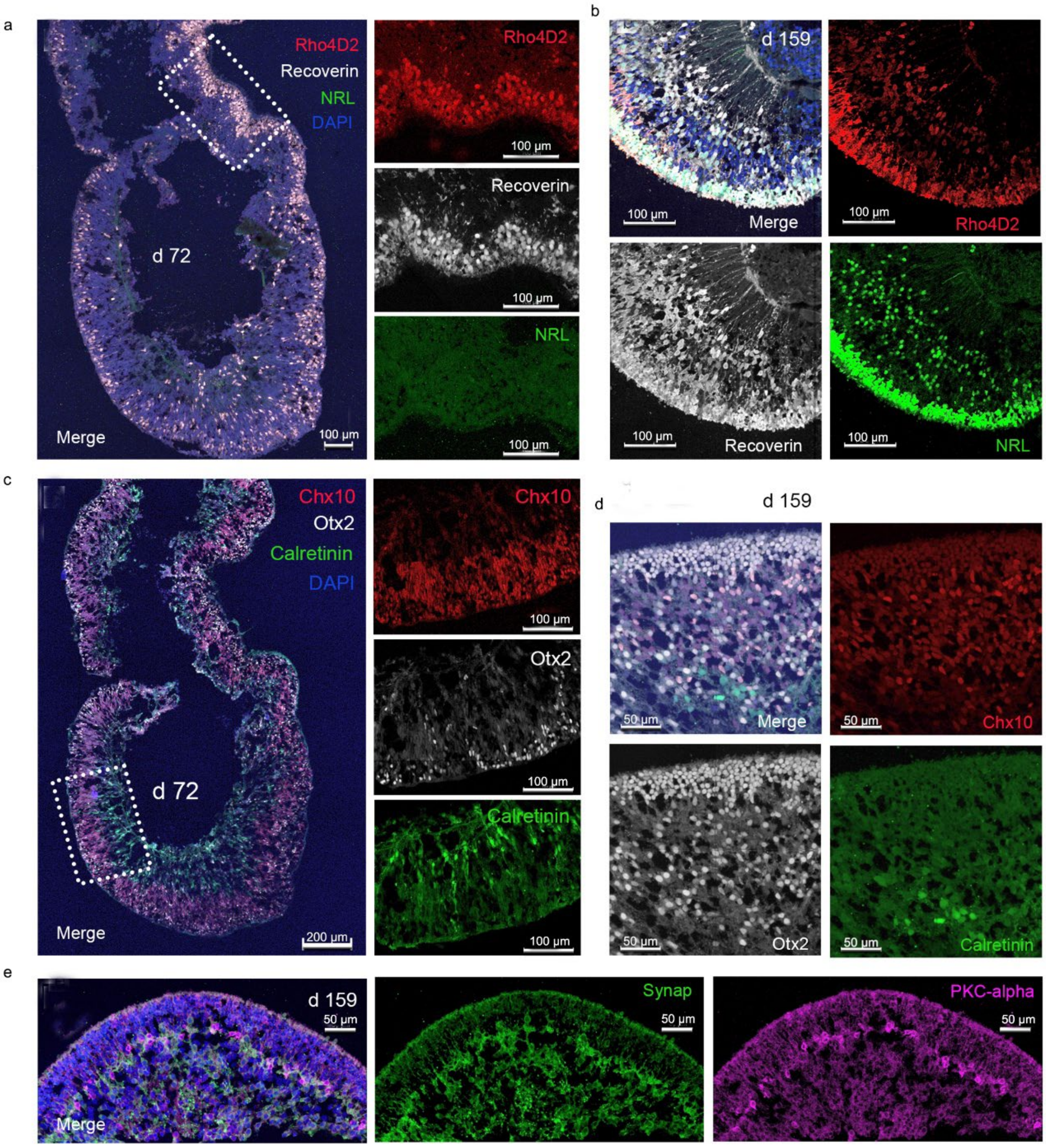

Fig. S1 Characterization of CRX-GFP retinal organoids. **a, b** Immunostaining image for rhodopsin (Rho4D2, red), recoverin (white), the rod-specific transcription factor neural retina leucine zipper (NRL), and nuclear stain DAPI (blue). **c, d** Immunostaining for ceh-10 homeodomain-containing homolog (Chx10, red), transcription factor orthodenticle homeobox 2 (Otx2, white), calretinin (green), and nuclear stain DAPI (blue). **e** Immunostaining of 159-d organoid for synaptophysin (Synap, green), the rod bipolar cell marker-protein kinase C alpha (PKCα; magenta), and nuclear stain DAPI (blue). Boxes indicate areas of enlargement. GFP green fluorescent protein, CRX cone-rod homeobox, DAPI 4′,6-diamidino-2-phenylindole

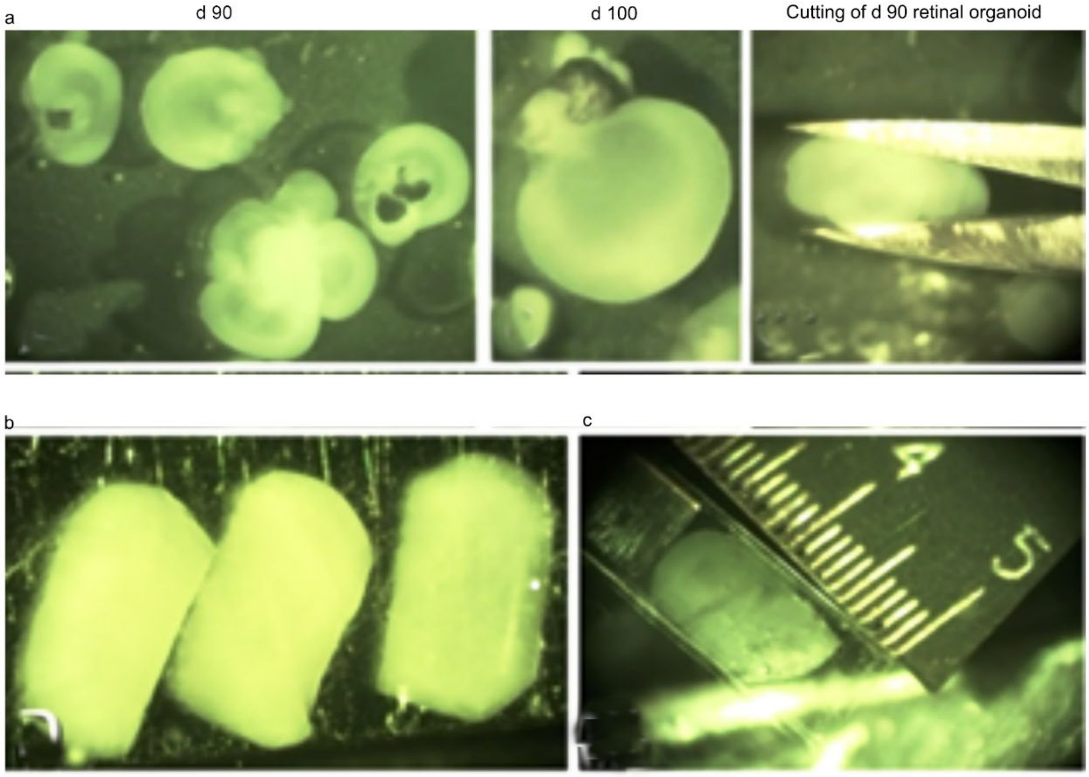

Fig. S2 Preparation of 3D retina organoids for transplantation. **a** Organoids at different developmental stages, d 90 and d 100 and cutting of d 90 organoid for making co-grafts. **b** Organoid cut into sheets. **c** Organoid sheets loaded into the nozzle of the transplant tool

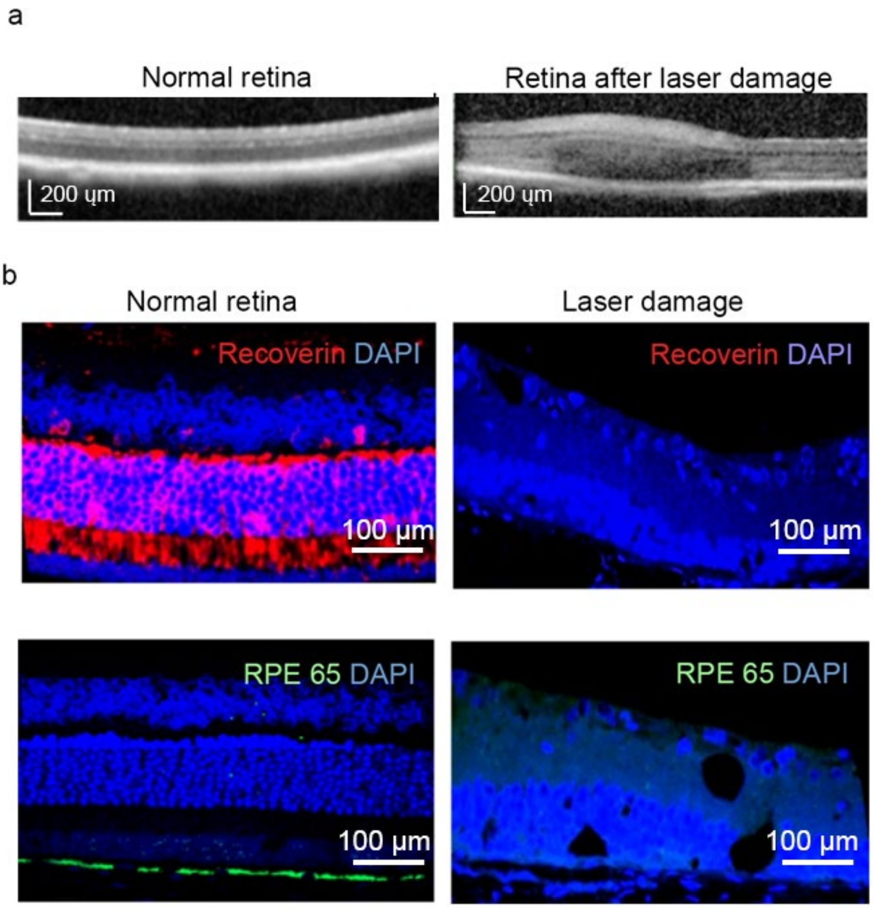

Fig. S3 Optical coherence tomography (OCT) and immunohistochemistry (IHC) of control and laser damaged retinas. **a** OCT image showing normal retina of a 2-month-old athymic nude rat and OCT of the retina immediately after laser damage showing loss of retinal layers [mainly outer nuclear layer (ONL) and RPE]. **b** Six-month-old normal retina showing recoverin (photoreceptor marker) staining and RPE 65 (RPE marker) staining (left panel). Six-month-old laser damaged retina in the right panel shows the absence of recoverin and RPE 65 staining. RPE 65 retinal pigment epithelium-specific 65 kD protein

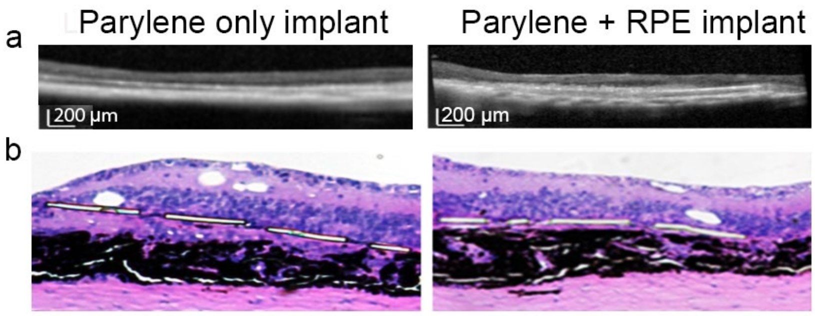

Fig. S4 Optical coherence tomography (OCT) and hematoxylin and eosin (HE) staining of parylene and retinal pigment epithelium (RPE) on parylene implanted retinas. **a** OCT image showing parylene and RPE on parylene implanted retinas after 6 months of implantation. **b** HE staining of parylene and RPE on parylene implanted retinas after 6 months of implantation

a

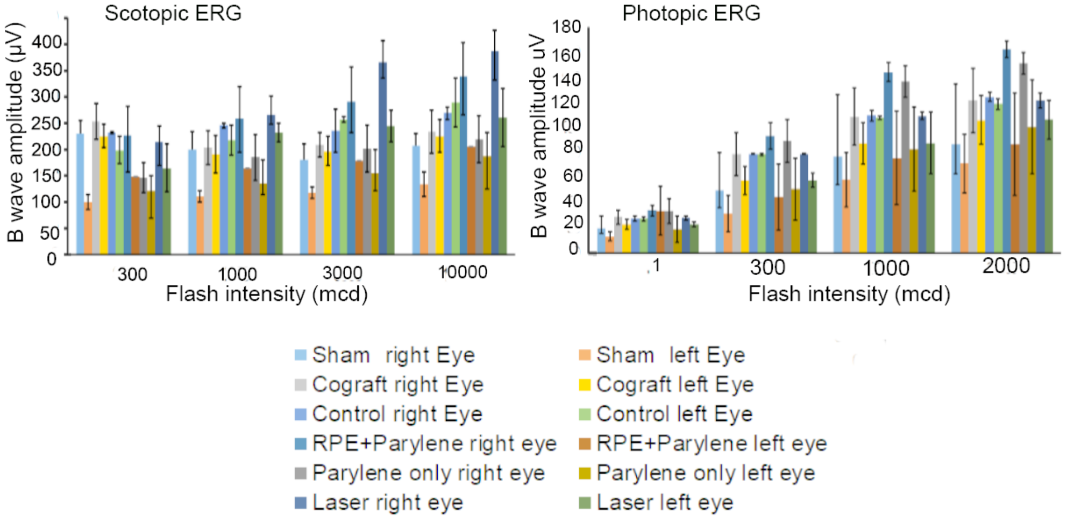

b

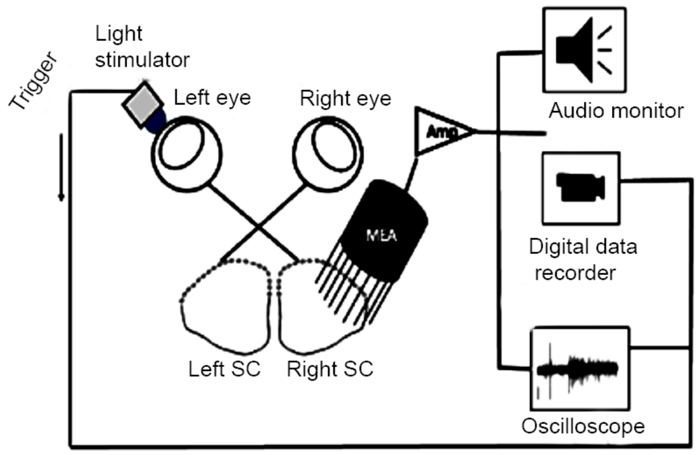

Fig. S5 Electroretinogram (ERG) data and superior colliculus (SC) electrophysiology setup. **a** ERG data at 7 months post-surgery. Despite the presence of laser-induced damage, no apparent changes were detected in the ERG recordings. Our study revealed the absence of obvious differences between the sham surgery (n = 8), laser-damaged control (n = 4), parylene only implant (n = 3), RPE + parylene implant (n = 9), and co-graft implanted group (n = 9), a phenomenon possibly attributed to the small size of the laser-damaged area. **b** SC electrophysiology set up. Schematic diagram showing electrophysiology setup used for mapping of the SC surface during full-field light stimulation. RPE retinal pigment epithelium, mcd millicandela

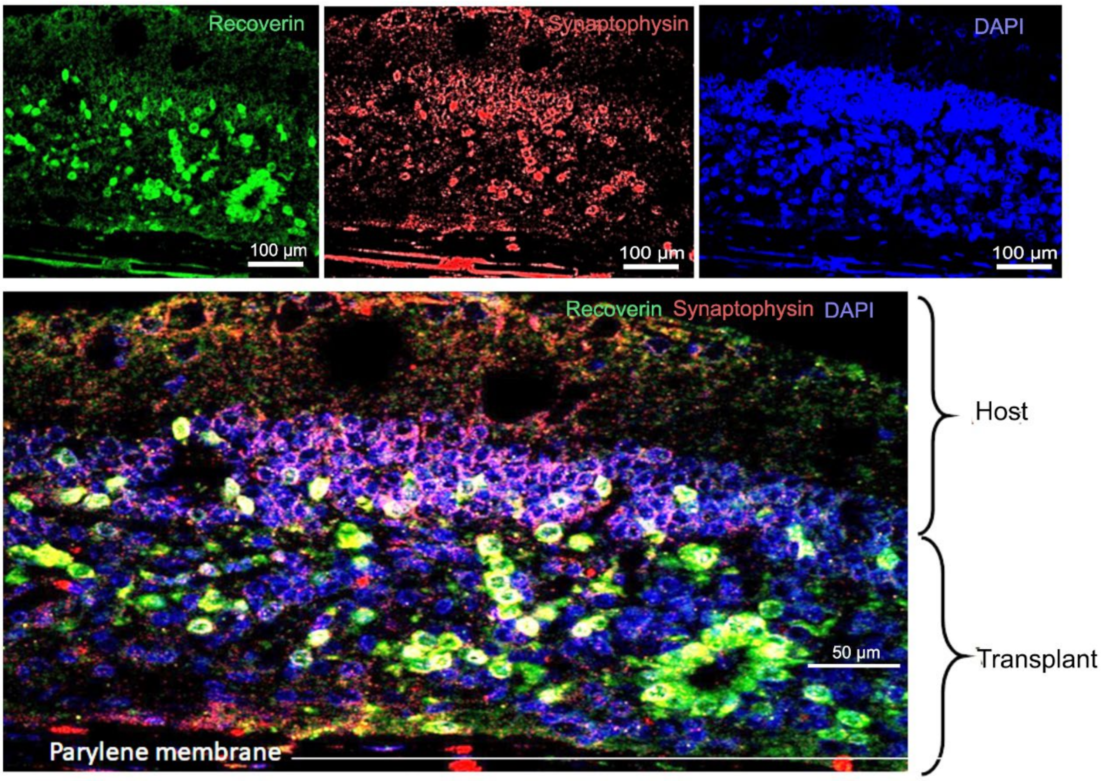

Fig. S6 Transplanted co-grafts expressing recoverin and synaptophysin. This image shows the only rosette structure found in the entire histology examination

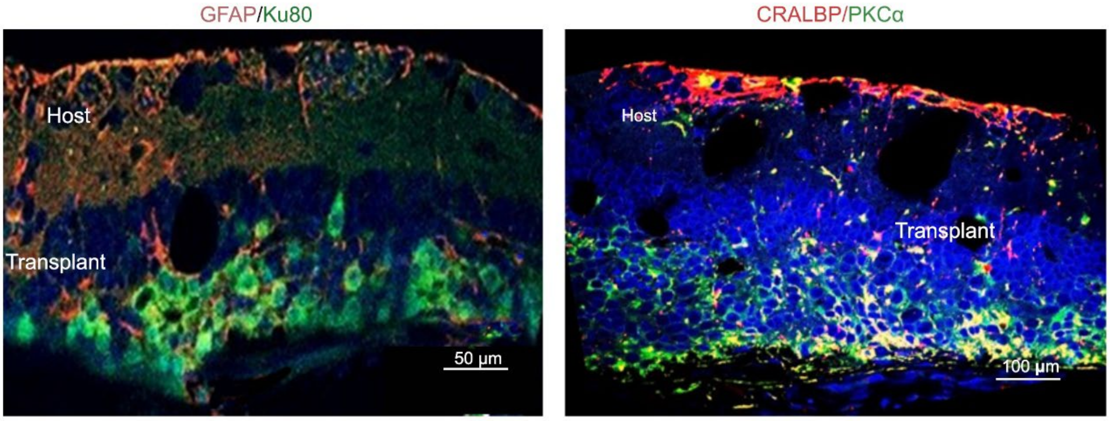

Fig. S7 Transplanted Co-grafts expressing glial fibrillary acidic protein (GFAP)/Ku80 (human nuclear marker), and cellular retinaldehyde binding protein (CRALPB; specific for Müller cells and RPE)/protein kinase C alpha (PKCα; human specific-Rod bipolar cell marker) after 7 months. Müller cells in the host are more reactive than in transplant. DAPI, blue. This monoclonal anti-CRALBP antibody stains the host stronger than the transplant
